# Supplementary material for: Discrimination performance in illness-death models with interval-censored disease data
Source: Stat Methods Med Res. 2026 Jan 29;35(3):469–87. doi: 10.1177/09622802251412855 (PMC13047238; doi:10.1177/09622802251412855)
Supplement: sj-pdf-1-smm-10.1177_09622802251412855 - Supplemental material for Discrimination performance in illness-death models with interval-censored disease data [file sj-pdf-1-smm-10.1177_09622802251412855.pdf]

Discrimination performance in illness-death models with interval-censored disease data

M. Spreafico, A.J. Rueten-Budde, H. Putter, and M. Fiocco

Supplementary Material

A Derivation of time-dependent AUC for a binary longitudinal marker

Throughout the article, a non-homogeneous Markov illness-death process is assumed, meaning the future evolution of the process $\{V(t), t > s\}$ depends only on the current state $V(s)$.

A.1 Incident/dynamic AUC

Let i, j be individuals, $X_i(t), X_j(t)$ their binary markers at time t, and T_i^D and T_j^D their death times. The incident/dynamic AUC is defined as

$$
\begin{aligned}
\mathrm{AUC}^{\mathrm{I/D}}(t) =& \Pr\left(X_i(t) > X_j(t) \mid T_i^D = t, T_j^D > t\right) + 0.5 \Pr\left(X_i(t) = X_j(t) \mid T_i^D = t, T_j^D > t\right) \\
=& \Pr\left(X_j(t) = 0 \mid T_j^D > t\right) \Pr\left(X_i(t) = 1 \mid T_i^D = t\right) \\
&+ 0.5 \cdot \big[\Pr\left(X_j(t) = 0 \mid T_j^D > t\right) \Pr\left(X_i(t) = 0 \mid T_i^D = t\right) + \\
&\qquad \Pr\left(X_j(t) = 1 \mid T_j^D > t\right) \Pr\left(X_i(t) = 1 \mid T_i^D = t\right) \big] \\
=& \left[1 - \Pr\left(X_j(t) = 1 \mid T_j^D > t\right)\right] \Pr\left(X_i(t) = 1 \mid T_i^D = t\right) \\
&+ 0.5 \cdot \Big\{ \left[1 - \Pr\left(X_j(t) = 1 \mid T_j^D > t\right)\right] \left[1 - \Pr\left(X_i(t) = 1 \mid T_i^D = t\right)\right] + \\
&\qquad \Pr\left(X_j(t) = 1 \mid T_j^D > t\right) \Pr\left(X_i(t) = 1 \mid T_i^D = t\right) \Big\} \\
=& \left[1 - \pi_1(t)\right] p(t) + 0.5 \left\{\left[1 - \pi_1(t)\right]\left[1 - p(t)\right] + \pi_1(t) p(t)\right\} \\
=& p(t) - \pi_1(t) p(t) + 0.5 \left[1 - p(t) - \pi_1(t) + \pi_1(t) p(t) + \pi_1(t) p(t)\right] \\
=& p(t) - \pi_1(t) p(t) + 0.5 - 0.5 p(t) - 0.5 \pi_1(t) + \pi_1(t) p(t) \\
=& 0.5 + 0.5 \left[p(t) - \pi_1(t)\right],
\end{aligned}
$$

where

$$
\pi_1(t) = \Pr\left(X_i(t) = 1 \mid T_i^D > t\right) \quad \text{and} \quad p(t) = \Pr\left(X_i(t-) = 1 \mid T_i^D = t\right).
$$

A.2 Cumulative/dynamic AUC

Let i, j be individuals, $X_i(s), X_j(s)$ their binary markers at time s, and T_i^D and T_j^D their death times. The cumulative/dynamic AUC is then

$$
\begin{aligned}
\mathrm{AUC}^{\mathrm{C/D}}(s,t) =& \Pr\left(X_i(s) > X_j(s) \mid s < T_i^D \le t, T_j^D > t\right) + 0.5 \Pr\left(X_i(s) = X_j(s) \mid s < T_i^D \le t, T_j^D > t\right) \\
=& \Pr\left(X_j(s) = 0 \mid T_j^D > t\right) \Pr\left(X_i(s) = 1 \mid s < T_i^D \le t\right) \\
&+ 0.5 \cdot \Big[\Pr\left(X_j(s) = 0 \mid T_j^D > t\right) \Pr\left(X_i(s) = 0 \mid s < T_i^D \le t\right) + \\
&\qquad \Pr\left(X_j(s) = 1 \mid T_j^D > t\right) \Pr\left(X_i(s) = 1 \mid s < T_i^D \le t\right) \Big] \\
=& \left[1 - \Pr\left(X_j(s) = 1 \mid T_j^D > t\right)\right] \Pr\left(X_i(s) = 1 \mid s < T_i^D \le t\right) \\
&+ 0.5 \cdot \Big\{ \left[1 - \Pr\left(X_j(s) = 1 \mid T_j^D > t\right)\right] \left[1 - \Pr\left(X_i(s) = 1 \mid s < T_i^D \le t\right)\right] + \\
&\qquad \Pr\left(X_j(s) = 1 \mid T_j^D > t\right) \Pr\left(X_i(s) = 1 \mid s < T_i^D \le t\right) \Big\} \\
=& \left[1 - \pi_1(s,t)\right] p(s,t) + 0.5 \left\{\left[1 - \pi_1(s,t)\right]\left[1 - p(s,t)\right] + \pi_1(s,t)p(s,t)\right\} \\
=& p(s,t) - \pi_1(s,t)p(s,t) + 0.5\left[1 - p(s,t) - \pi_1(s,t) + \pi_1(s,t)p(s,t) + \pi_1(s,t)p(s,t)\right] \\
=& p(s,t) - \pi_1(s,t)p(s,t) + 0.5 - 0.5p(s,t) - 0.5\pi_1(s,t) + \pi_1(s,t)p(s,t) \\
=& 0.5 + 0.5\left[p(s,t) - \pi_1(s,t)\right],
\end{aligned}
$$

where

$$
\pi_1(s,t) = \Pr\left(X_j(s) = 1 \mid T_j^D > t\right) \quad \text{and} \quad p(s,t) = \Pr\left(X_i(s) = 1 \mid s < T_i^D \le t\right).
$$

B Weibull illness-death model

The transition hazards from state h to state l are set to be Weibull distributed and defined as follows:

$$\lambda_{hl}(t) = \alpha_{hl} k t^{k-1}, \tag{1}$$

where k is the common shape parameter and α_{hl} are transition-specific rate parameters. Let

$$S_0(t) = \exp\left\{-(\alpha_{01} + \alpha_{02})t^k\right\}$$

and

$$S_1(t) = \exp\left\{-\alpha_{12} t^k\right\}.$$

Then, the transition probabilities are equal to

$$P_{00}(u,t) = \frac{S_0(t)}{S_0(u)},$$

$$P_{11}(u,t) = \frac{S_1(t)}{S_1(u)},$$

$$P_{01}(u,t) = \begin{cases} \frac{\alpha_{01}}{\alpha_{01}+\alpha_{02}-\alpha_{12}} \left(\frac{S_1(t)}{S_1(u)} - \frac{S_0(t)}{S_0(u)}\right) & \text{if } \alpha_{01}+\alpha_{02}-\alpha_{12} \neq 0 \\ \alpha_{01} \left(\frac{S_1(t)}{S_1(u)} t^k - \frac{S_0(t)}{S_0(u)} u^k\right) & \text{otherwise (note: } S_1(t) = S_0(t)), \end{cases}$$

$$P_{02}^0(u,t) = \frac{\alpha_{02}}{\alpha_{01}+\alpha_{02}} \left(1 - \frac{S_0(t)}{S_0(u)}\right),$$

$$P_{02}^1(u,t) = \begin{cases} \frac{\alpha_{01}}{\alpha_{01}+\alpha_{02}} \left(1 - \frac{S_0(t)}{S_0(u)}\right) - \frac{\alpha_{01}}{\alpha_{01}+\alpha_{02}-\alpha_{12}} \left(\frac{S_1(t)}{S_1(u)} - \frac{S_0(t)}{S_0(u)}\right) & \text{if } \alpha_{01}+\alpha_{02}-\alpha_{12} \neq 0 \\ \frac{\alpha_{01}}{\alpha_{01}+\alpha_{02}} \left(1 - \frac{S_0(t)}{S_0(u)}\right) - \alpha_{01} \frac{S_0(t)}{S_0(u)} \left(t^k - u^k\right) & \text{otherwise,} \end{cases}$$

$$P_{02}(u,t) = P_{02}^0(u,t) + P_{02}^1(u,t) =$$

$$= 1 - \frac{\alpha_{02}-\alpha_{12}}{\alpha_{01}+\alpha_{02}-\alpha_{12}} \cdot \frac{S_0(t)}{S_0(u)} - \frac{\alpha_{01}}{\alpha_{01}+\alpha_{02}-\alpha_{12}} \cdot \frac{S_1(t)}{S_1(u)},$$

and

$$P_{12}(u,t) = 1 - \frac{S_1(t)}{S_1(u)},$$

where $P_{hl}(u,t)$ is the conditional probability of being in state l, $(l = 0,1,2)$ at time t given in state h, $(h = 0,1)$ at time u.

These transition probabilities can be used to calculate the time-specific incident/dynamic and cumulative/dynamic AUC using Manuscript Equations (9) and (15), respectively. For the R code, see the file `functions/weibull_auc.R` available at http://github.com/mspreafico/auc-idmIC.

C Simulation study: Results for all scenarios

C.1 Estimated models

Table C.1: Bias, empirical standard error (SE), and root mean square error (RMSE) for the effect of disease (yes vs no) for the Cox and piecewise constant (PW-const) models under scenarios A to R in Manuscript Table 1.

Scenario	Model	Mean(β)	exp(mean(β))	SE(β)	Bias(β)	RMSE(β)
Truth		2.42	11.20			
A	Cox ROC	2.35	10.44	0.09	-0.07	0.11
	Cox prob	2.35	10.44	0.09	-0.07	0.11
	PW-const	2.43	11.32	0.09	0.01	0.09
B	Cox ROC	2.29	9.91	0.10	-0.12	0.16
	Cox prob	2.29	9.91	0.10	-0.12	0.16
	PW-const	2.48	11.90	0.10	0.06	0.12
C	Cox ROC	2.24	9.37	0.11	-0.18	0.21
	Cox prob	2.24	9.37	0.11	-0.18	0.21
	PW-const	2.41	11.11	0.12	-0.01	0.12
D	Cox ROC	2.35	10.45	0.09	-0.07	0.11
	Cox prob	2.35	10.45	0.09	-0.07	0.11
	PW-const	2.45	11.59	0.08	0.03	0.09
E	Cox ROC	2.31	10.05	0.09	-0.11	0.14
	Cox prob	2.31	10.05	0.09	-0.11	0.14
	PW-const	2.50	12.19	0.10	0.08	0.13
F	Cox ROC	2.25	9.47	0.10	-0.17	0.20
	Cox prob	2.25	9.47	0.10	-0.17	0.20
	PW-const	2.43	11.34	0.11	0.01	0.11
G	Cox ROC	2.34	10.43	0.07	-0.07	0.10
	Cox prob	2.34	10.43	0.07	-0.07	0.10
	PW-const	2.42	11.30	0.06	0.01	0.06
H	Cox ROC	2.29	9.91	0.07	-0.12	0.14
	Cox prob	2.29	9.91	0.07	-0.12	0.14
	PW-const	2.47	11.88	0.07	0.06	0.09
I	Cox ROC	2.24	9.38	0.08	-0.18	0.20
	Cox prob	2.24	9.38	0.08	-0.18	0.20
	PW-const	2.41	11.09	0.08	-0.01	0.08
J	Cox ROC	2.34	10.40	0.06	-0.07	0.10
	Cox prob	2.34	10.40	0.06	-0.07	0.10
	PW-const	2.44	11.52	0.06	0.03	0.07
K	Cox ROC	2.30	10.00	0.06	-0.11	0.13
	Cox prob	2.30	10.00	0.06	-0.11	0.13
	PW-const	2.49	12.10	0.07	0.08	0.10
L	Cox ROC	2.24	9.40	0.07	-0.17	0.19
	Cox prob	2.24	9.40	0.07	-0.17	0.19
	PW-const	2.42	11.24	0.08	0.00	0.08
M	Cox ROC	2.34	10.40	0.15	-0.07	0.17
	Cox prob	2.34	10.40	0.15	-0.07	0.17
	PW-const	2.43	11.34	0.14	0.01	0.14
N	Cox ROC	2.29	9.91	0.16	-0.12	0.20
	Cox prob	2.29	9.91	0.16	-0.12	0.20
	PW-const	2.48	11.95	0.16	0.06	0.17
O	Cox ROC	2.24	9.43	0.18	-0.17	0.25
	Cox prob	2.24	9.43	0.18	-0.17	0.25
	PW-const	2.42	11.24	0.19	0.00	0.19
P	Cox ROC	2.34	10.43	0.15	-0.07	0.16
	Cox prob	2.34	10.43	0.15	-0.07	0.16
	PW-const	2.45	11.63	0.14	0.04	0.14
Q	Cox ROC	2.31	10.04	0.15	-0.11	0.19
	Cox prob	2.31	10.04	0.15	-0.11	0.19
	PW-const	2.51	12.25	0.15	0.09	0.18
R	Cox ROC	2.24	9.42	0.16	-0.17	0.23
	Cox prob	2.24	9.42	0.16	-0.17	0.23
	PW-const	2.43	11.35	0.16	0.01	0.16

Table C.2: Number of invalid estimations of AUC$^{I/D}$ and AUC$^{C/D}$ from 1 year based on $n_{sim} = 1000$ data sets for the M-spline model under scenarios A to R.

Scenario	invalid AUC$^{I/D}$	invalid AUC$^{C/D}$
A	379	381
B	442	444
C	555	560
D	17	17
E	34	34
F	88	89
G	201	201
H	292	296
I	378	381
J	1	1
K	7	7
L	19	19
M	319	315
N	299	295
O	190	188
P	752	751
Q	776	773
R	639	638

C.2 Additional results for scenarios A–F ($N = 1000$)

Table C.3: Bias, empirical standard error (SE), and root mean square error (RMSE) for time-specific incident/dynamic AUC at 1, 3, 5 years from the Cox model under scenarios A to F estimated via the `risksetAUC` function from the `risksetROC` R-package.

		AUC$^{I/D}$(1) = 0.71			AUC$^{I/D}$(3) = 0.72			AUC$^{I/D}$(5) = 0.72		
Scenario	Model	Bias	SE	RMSE	Bias	SE	RMSE	Bias	SE	RMSE
A	Cox ROC	-0.04	0.02	0.04	-0.02	0.01	0.03	-0.02	0.01	0.02
B	Cox ROC	-0.07	0.02	0.08	-0.04	0.01	0.04	-0.03	0.01	0.04
C	Cox ROC	-0.21	0.00	0.21	-0.07	0.02	0.08	-0.06	0.02	0.06
D	Cox ROC	-0.04	0.02	0.04	-0.02	0.01	0.02	-0.02	0.01	0.02
E	Cox ROC	-0.07	0.02	0.08	-0.04	0.01	0.04	-0.03	0.02	0.03
F	Cox ROC	-0.21	0.00	0.21	-0.07	0.02	0.08	-0.06	0.02	0.06

Abbreviations: AUC$^{I/D}(t)$, incident/dynamic AUC at year t

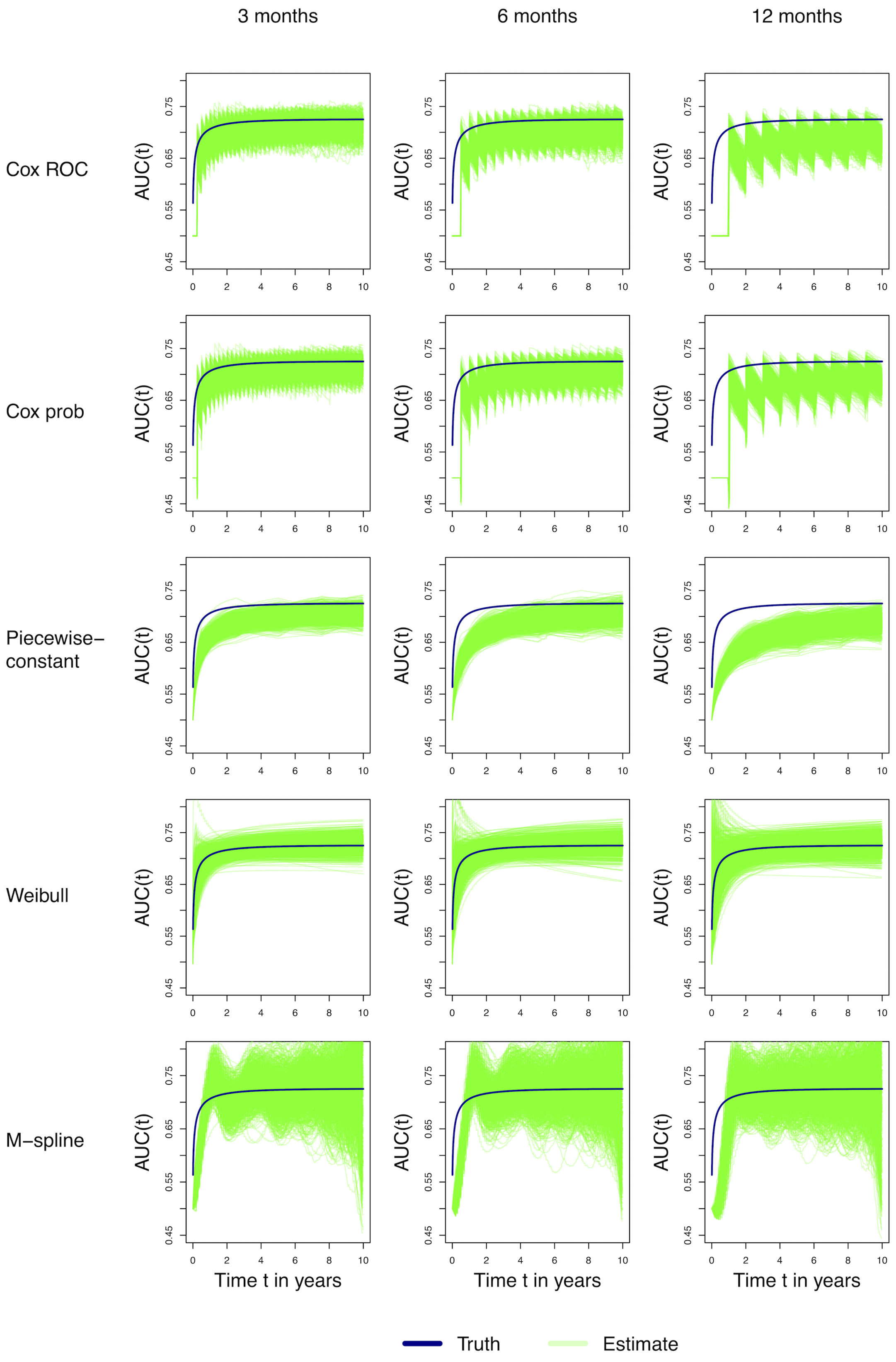

Figure C.1: Estimated time-specific incident/dynamic AUC for scenario D (3 months; left panels), E (6 months; middle panels) and F (12 months; right panels) using different models (Cox, PW-const, Weibull, M-spline). The x-axis represents time t in years; the y-axis represents $\widehat{\text{AUC}}^{\text{I/D}}(t)$. The blue line in each panel represents the true values over time. Estimates for *Cox ROC* (first row) are based on `risksetAUC` function for Cox model. Estimates for *Cox prob* are based on transition probabilities of Cox model via `mstate` package.

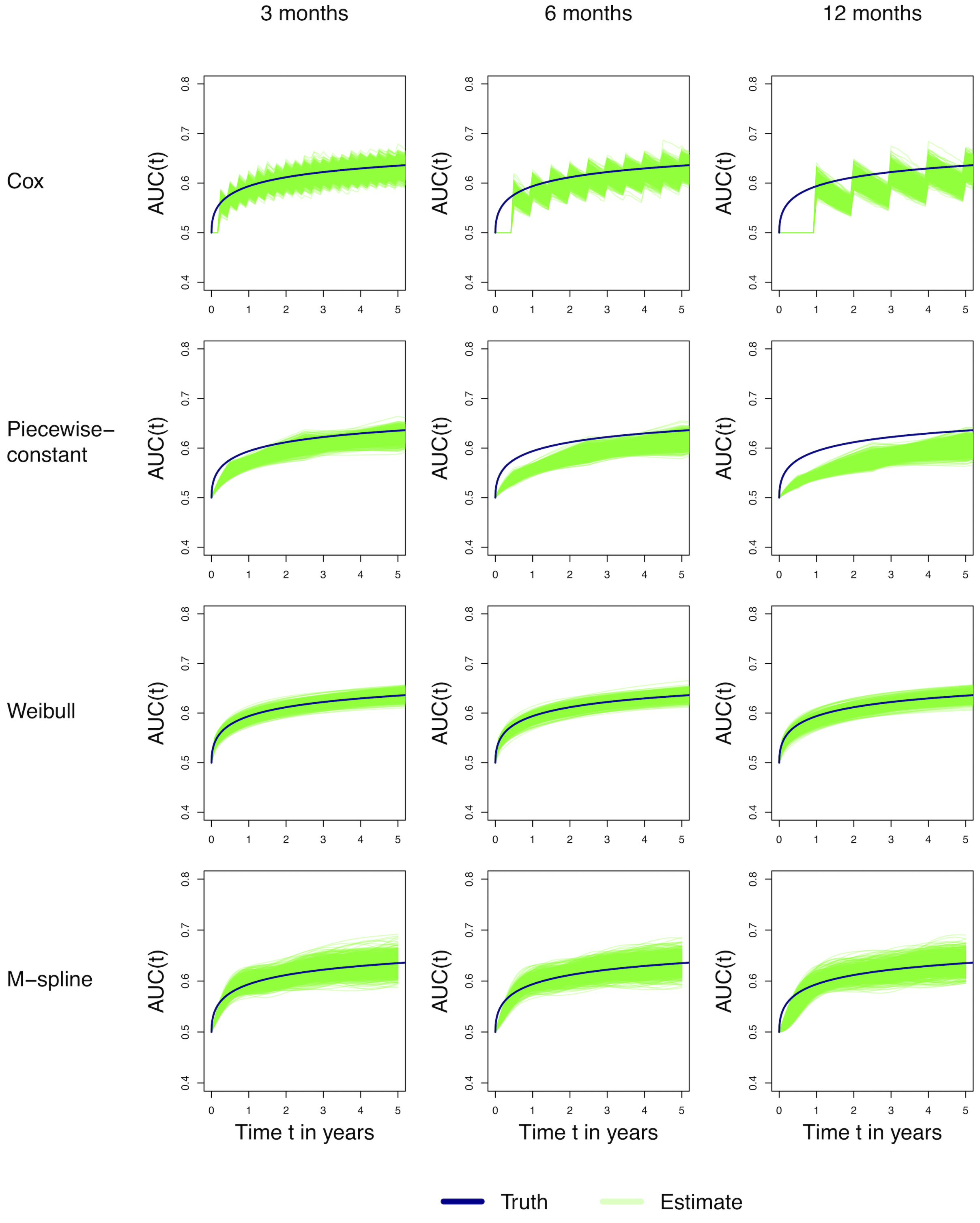

Figure C.2: Estimated time-specific cumulative/dynamic AUC for scenario D (3 months; left panels), E (6 months; middle panels) and F (12 months; right panels) using different models (Cox, PW-const, Weibull, M-spline). The x-axis represents the prediction time t in years. The prediction window is set to 5 years, so the y-axis represents $\widehat{\text{AUC}}^{\text{C/D}}(t, t+5)$. The blue line in each panel represents the true values over time.

C.3 Results for scenarios G–L ($N = 2000$)

Table C.4: Estimated bias, empirical standard error (SE), and root mean square error (RMSE) for time-specific incident/dynamic AUC at 1, 3, 5 years under scenarios G to L.

		$\text{AUC}^{\text{I/D}}(1) = 0.71$			$\text{AUC}^{\text{I/D}}(3) = 0.72$			$\text{AUC}^{\text{I/D}}(5) = 0.72$		
Scenario	Model	Bias	SE	RMSE	Bias	SE	RMSE	Bias	SE	RMSE
G	Cox ROC	-0.04	0.01	0.04	-0.02	0.01	0.02	-0.02	0.01	0.02
	Cox prob	-0.04	0.01	0.04	-0.02	0.01	0.02	-0.02	0.01	0.02
	PW-const	-0.05	0.01	0.05	-0.03	0.01	0.03	-0.02	0.01	0.03
	Weibull	0.00	0.01	0.01	0.00	0.01	0.01	0.00	0.01	0.01
	M-spline	0.02	0.02	0.03	0.00	0.02	0.02	-0.01	0.02	0.03
H	Cox ROC	-0.07	0.01	0.08	-0.04	0.01	0.04	-0.03	0.01	0.03
	Cox prob	-0.07	0.01	0.08	-0.04	0.01	0.04	-0.03	0.01	0.03
	PW-const	-0.06	0.01	0.06	-0.04	0.01	0.04	-0.03	0.01	0.03
	Weibull	-0.01	0.01	0.01	0.00	0.01	0.01	0.00	0.01	0.01
	M-spline	0.01	0.02	0.02	-0.01	0.02	0.02	-0.01	0.03	0.03
I	Cox ROC	-0.21	0.00	0.21	-0.07	0.01	0.07	-0.06	0.01	0.06
	Cox prob	-0.21	0.00	0.21	-0.07	0.01	0.07	-0.06	0.01	0.06
	PW-const	-0.09	0.01	0.09	-0.06	0.01	0.06	-0.05	0.01	0.05
	Weibull	-0.01	0.02	0.02	0.00	0.01	0.01	0.00	0.01	0.01
	M-spline	-0.04	0.03	0.05	0.00	0.02	0.02	0.00	0.03	0.03
J	Cox ROC	-0.04	0.01	0.04	-0.02	0.01	0.02	-0.02	0.01	0.02
	Cox prob	-0.04	0.01	0.04	-0.02	0.01	0.02	-0.02	0.01	0.02
	PW-const	-0.05	0.01	0.05	-0.03	0.01	0.03	-0.02	0.01	0.03
	Weibull	0.00	0.01	0.01	0.00	0.01	0.01	0.00	0.01	0.01
	M-spline	0.02	0.01	0.03	-0.01	0.02	0.02	-0.01	0.02	0.03
K	Cox ROC	-0.07	0.01	0.07	-0.04	0.01	0.04	-0.03	0.01	0.03
	Cox prob	-0.07	0.01	0.07	-0.04	0.01	0.04	-0.03	0.01	0.03
	PW-const	-0.06	0.01	0.06	-0.04	0.01	0.04	-0.03	0.01	0.03
	Weibull	-0.01	0.01	0.01	0.00	0.01	0.01	0.00	0.01	0.01
	M-spline	0.01	0.02	0.02	-0.01	0.02	0.02	0.00	0.02	0.03
L	Cox ROC	-0.21	0.00	0.21	-0.07	0.01	0.07	-0.06	0.01	0.06
	Cox prob	-0.21	0.00	0.21	-0.07	0.01	0.07	-0.06	0.01	0.06
	PW-const	-0.09	0.01	0.09	-0.06	0.01	0.06	-0.05	0.01	0.05
	Weibull	-0.01	0.02	0.02	0.00	0.01	0.01	0.00	0.01	0.01
	M-spline	-0.04	0.03	0.05	0.00	0.02	0.02	0.00	0.03	0.03

Abbreviations: $\text{AUC}^{\text{I/D}}(t)$, incident/dynamic AUC at year t; PW-const, piecewise-constant model; *Cox ROC* estimates are obtained via the `risksetAUC` function from the `risksetROC` package; *Cox prob* estimates are based on transition probabilities of Cox model through `mstate` package.

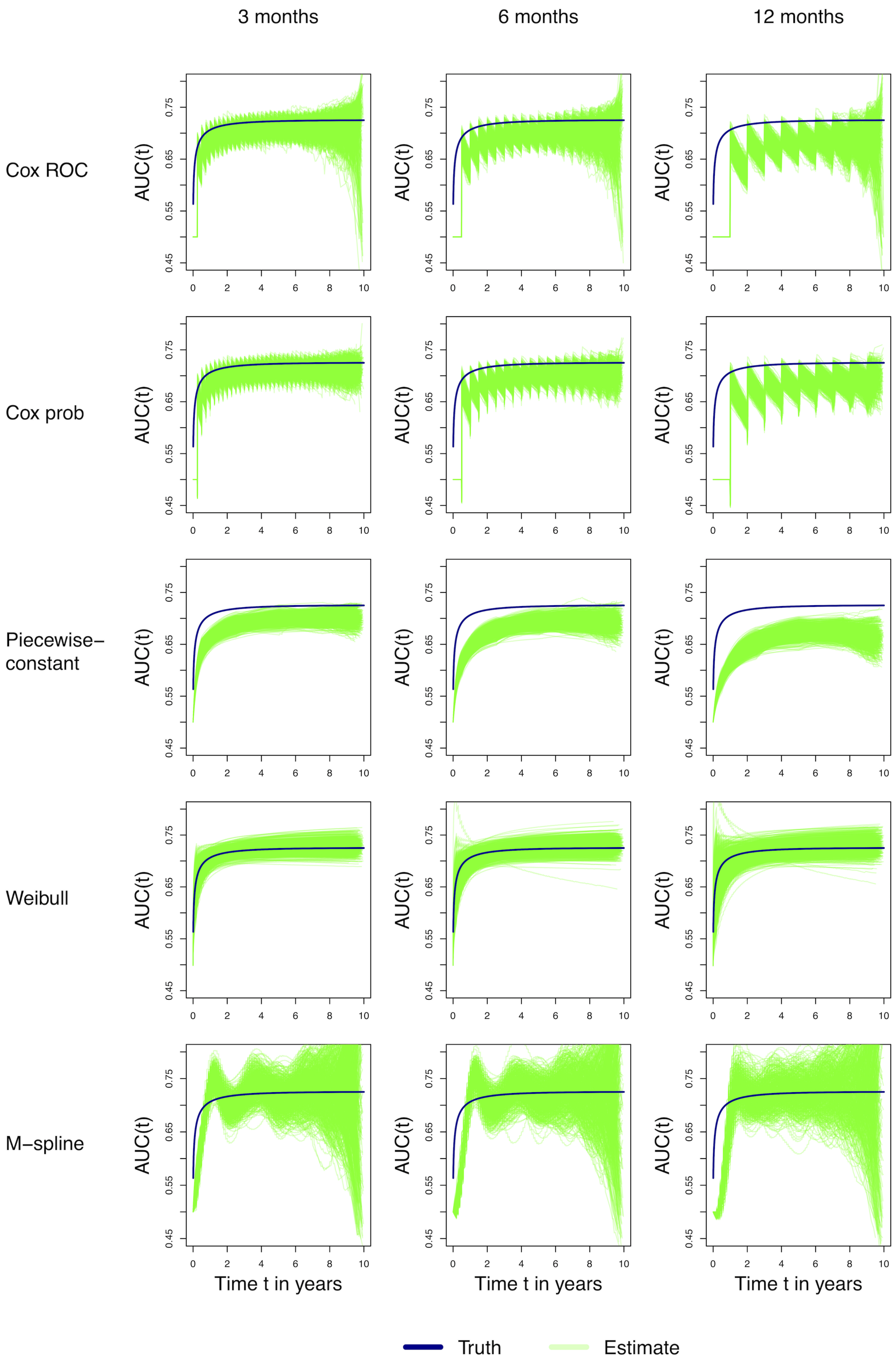

Figure C.3: Estimated time-specific incident/dynamic AUC for scenario G (3 months; left panels), H (E months; middle panels) and I (12 months; right panels) using different models (Cox, PW-const, Weibull, M-spline). The x-axis represents time t in years; the y-axis represents $\widehat{\mathrm{AUC}}^{\mathrm{I/D}}(t)$. The blue line in each panel represents the true values over time. Estimates for *Cox ROC* (first row) are based on `risksetAUC` function for Cox model. Estimates for *Cox prob* are based on transition probabilities of Cox model via `mstate` package.

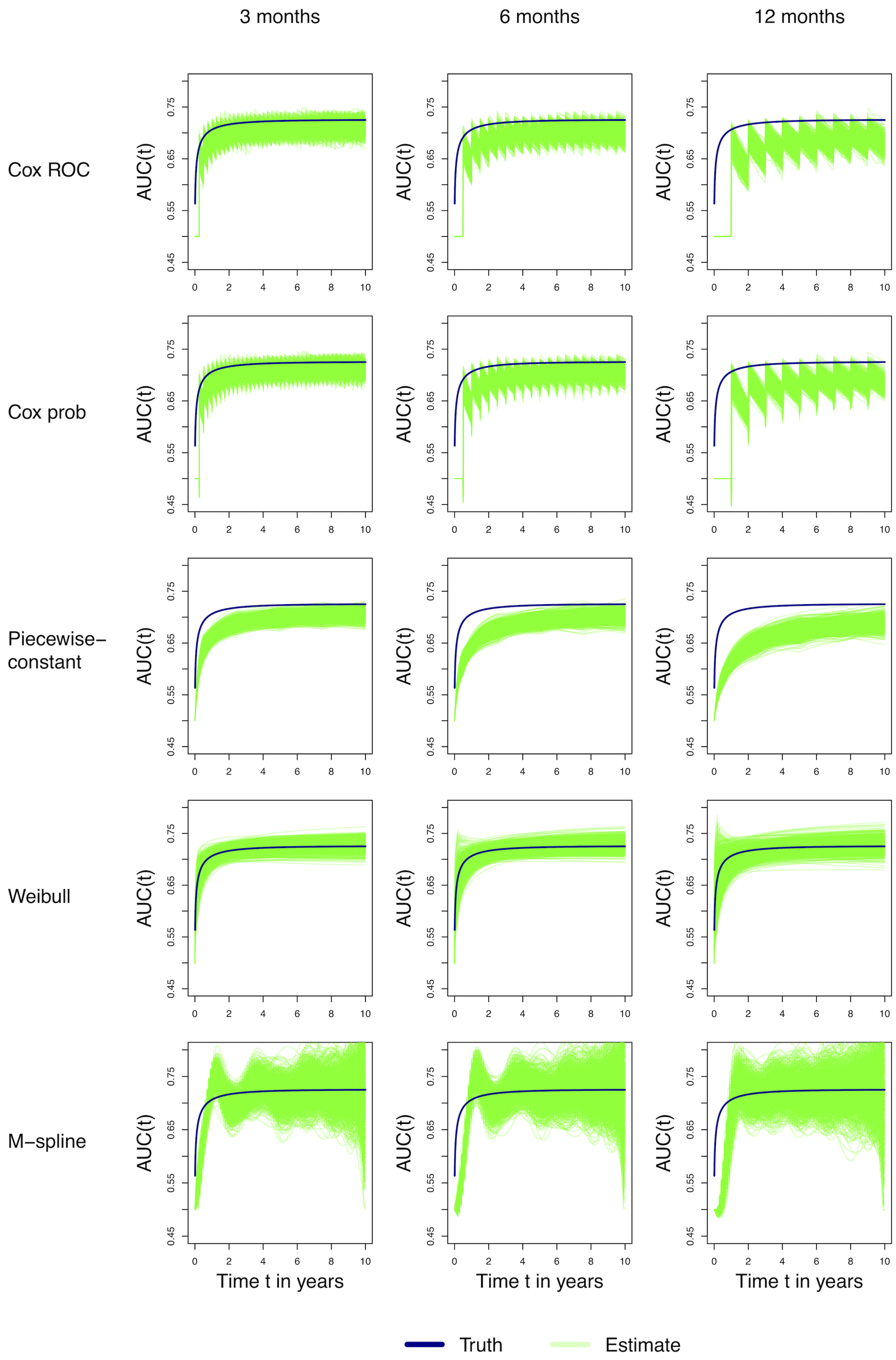

Figure C.4: Estimated time-specific incident/dynamic AUC for scenario J (3 months; left panels), K (6 months; middle panels) and L (12 months; right panels) using different models (Cox, PW-const, Weibull, M-spline). The x-axis represents time t in years; the y-axis represents $\widehat{\text{AUC}}^{\text{I/D}}(t)$. The blue line in each panel represents the true values over time. Estimates for *Cox ROC* (first row) are based on `risksetAUC` function for Cox model. Estimates for *Cox prob* are based on transition probabilities of Cox model via `mstate` package.

Table C.5: Estimated bias, empirical standard error (SE), and root mean square error (RMSE) for time-specific cumulative/dynamic AUC for prediction time 1, 3, 5 years and prediction window of 5 years under scenarios G to L.

		$\text{AUC}^{C/D}(1,6) = 0.59$			$\text{AUC}^{C/D}(3,7) = 0.62$			$\text{AUC}^{C/D}(5,8) = 0.64$		
Scenario	Model	Bias	SE	RMSE	Bias	SE	RMSE	Bias	SE	RMSE
G	Cox	0.00	0.01	0.01	0.00	0.01	0.01	0.00	0.01	0.01
	PW-const	-0.02	0.00	0.02	-0.01	0.01	0.01	-0.01	0.01	0.01
	Weibull	0.00	0.00	0.01	0.00	0.00	0.00	0.00	0.01	0.01
	M-spline	0.00	0.01	0.01	0.00	0.01	0.01	0.00	0.01	0.01
H	Cox	0.00	0.01	0.01	0.00	0.01	0.01	0.00	0.01	0.01
	PW-const	-0.04	0.00	0.04	-0.02	0.01	0.02	-0.02	0.01	0.02
	Weibull	0.00	0.00	0.01	0.00	0.01	0.01	0.00	0.01	0.01
	M-spline	0.00	0.01	0.01	0.00	0.01	0.01	0.00	0.01	0.01
I	Cox	0.00	0.01	0.01	0.00	0.01	0.01	0.00	0.01	0.01
	PW-const	-0.05	0.00	0.05	-0.04	0.01	0.04	-0.03	0.01	0.03
	Weibull	-0.01	0.01	0.01	0.00	0.01	0.01	0.00	0.01	0.01
	M-spline	0.00	0.01	0.01	0.00	0.01	0.01	0.00	0.01	0.01
J	Cox	0.00	0.01	0.01	0.00	0.01	0.01	0.00	0.01	0.01
	PW-const	-0.02	0.00	0.02	-0.01	0.01	0.01	-0.01	0.01	0.01
	Weibull	0.00	0.00	0.01	0.00	0.00	0.00	0.00	0.00	0.00
	M-spline	0.00	0.01	0.01	0.00	0.01	0.01	0.00	0.01	0.01
K	Cox	0.00	0.01	0.01	0.00	0.01	0.01	0.00	0.01	0.01
	PW-const	-0.04	0.00	0.04	-0.02	0.01	0.03	-0.02	0.01	0.02
	Weibull	0.00	0.00	0.01	0.00	0.00	0.00	0.00	0.01	0.01
	M-spline	0.00	0.01	0.01	0.00	0.01	0.01	0.00	0.01	0.01
L	Cox	0.00	0.01	0.01	0.00	0.01	0.01	0.00	0.01	0.01
	PW-const	-0.05	0.00	0.05	-0.04	0.01	0.04	-0.03	0.01	0.03
	Weibull	-0.01	0.01	0.01	0.00	0.01	0.01	0.00	0.01	0.01
	M-spline	0.00	0.01	0.01	0.00	0.01	0.01	0.00	0.01	0.01

Abbreviations: $\text{AUC}^{C/D}(t, t+5)$, cumulative/dynamic AUC for prediction time t in years and prediction window of 5 years

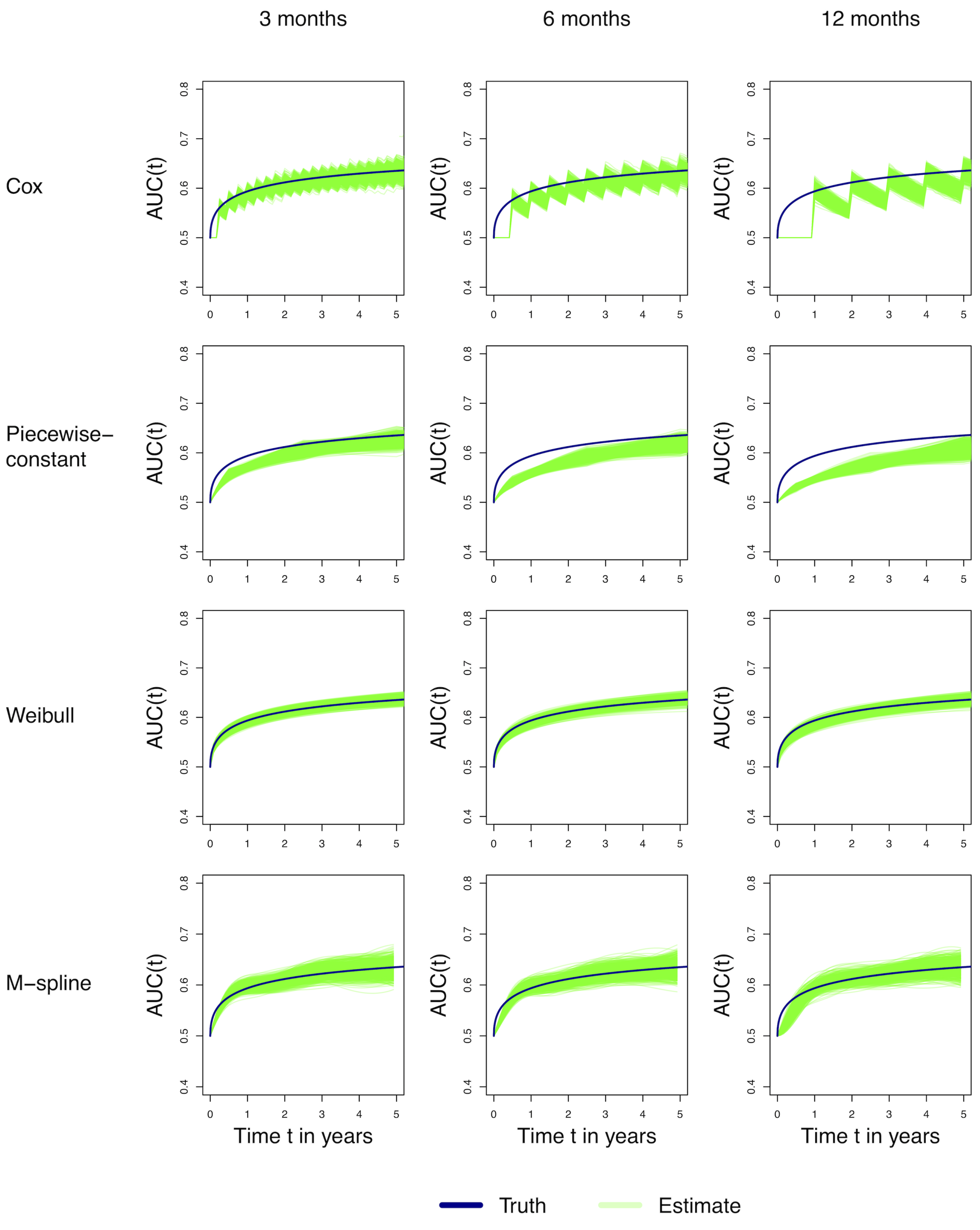

Figure C.5: Estimated time-specific cumulative/dynamic AUC for scenario G (3 months; left panels), H (6 months; middle panels) and I (12 months; right panels) using different models (Cox, PW-const, Weibull, M-spline). The x-axis represents the prediction time t in years. The prediction window is set to 5 years, so the y-axis represents $\widehat{\mathrm{AUC}}^{\mathrm{C/D}}(t, t+5)$. The blue line in each panel represents the true values over time.

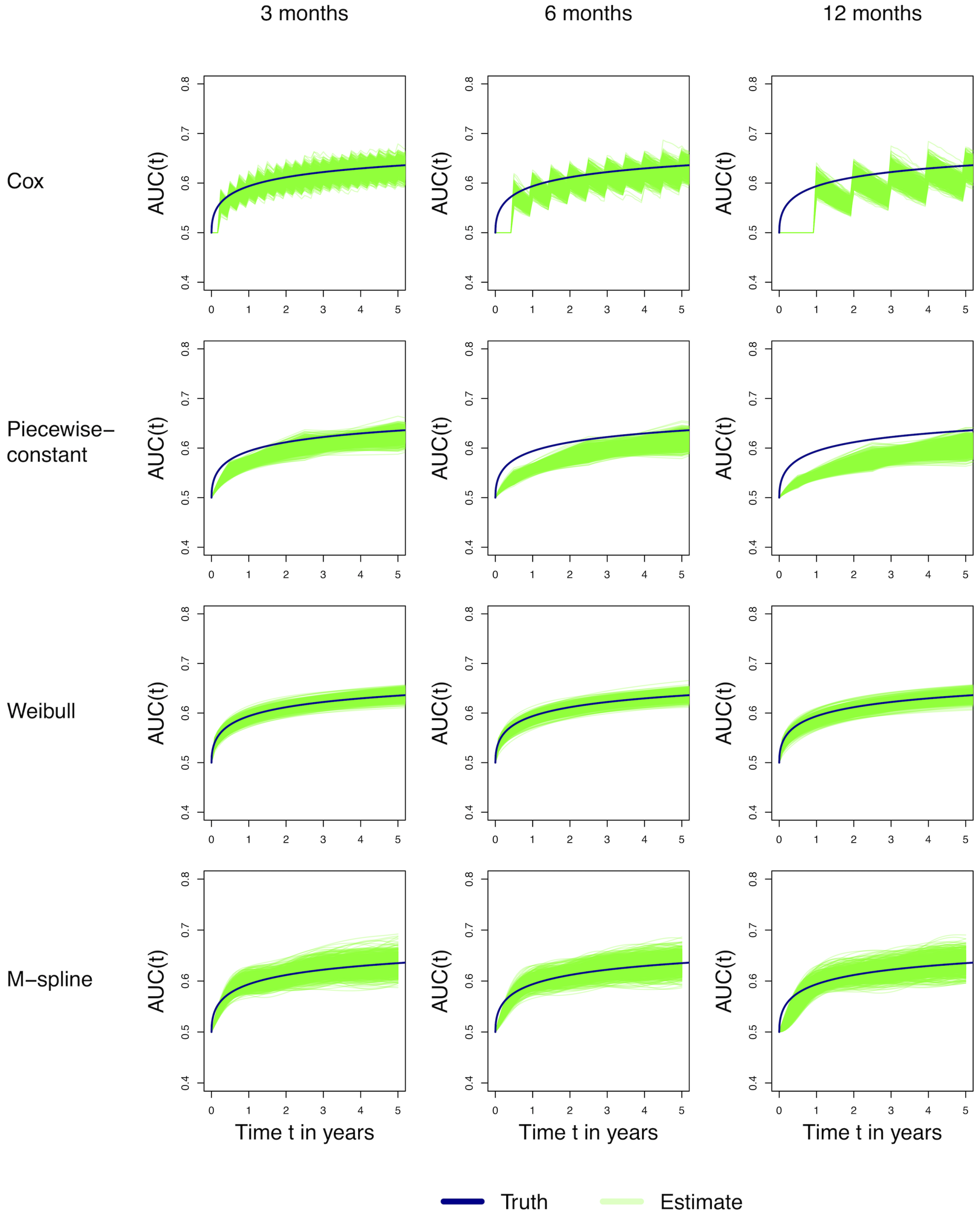

Figure C.6: Estimated time-specific cumulative/dynamic AUC for scenario J (3 months; left panels), K (6 months; middle panels) and L (12 months; right panels) using different models (Cox, PW-const, Weibull, M-spline). The x-axis represents the prediction time t in years. The prediction window is set to 5 years, so the y-axis represents $\widehat{\mathrm{AUC}}^{\mathrm{C/D}}(t, t+5)$. The blue line in each panel represents the true values over time.

C.4 Results for scenarios M–R ($N = 400$)

Table C.6: Estimated bias, empirical standard error (SE), and root mean square error (RMSE) for time-specific incident/dynamic AUC at 1, 3, 5 years under scenarios M to R.

		$\text{AUC}^{\text{I/D}}(1) = 0.71$			$\text{AUC}^{\text{I/D}}(3) = 0.72$			$\text{AUC}^{\text{I/D}}(5) = 0.72$		
Scenario	Model	Bias	SE	RMSE	Bias	SE	RMSE	Bias	SE	RMSE
M	Cox ROC	-0.04	0.03	0.04	-0.02	0.02	0.03	-0.02	0.02	0.03
	Cox prob	-0.04	0.02	0.04	-0.02	0.02	0.03	-0.02	0.02	0.03
	PW-const	-0.05	0.02	0.05	-0.03	0.02	0.04	-0.03	0.02	0.03
	Weibull	-0.00	0.02	0.02	0.00	0.02	0.02	0.00	0.02	0.02
	M-spline	0.01	0.03	0.04	-0.01	0.04	0.04	-0.00	0.05	0.05
N	Cox ROC	-0.08	0.03	0.08	-0.04	0.02	0.05	-0.03	0.02	0.04
	Cox prob	-0.07	0.02	0.08	-0.04	0.02	0.04	-0.03	0.02	0.04
	PW-const	-0.07	0.02	0.07	-0.04	0.02	0.04	-0.03	0.02	0.04
	Weibull	-0.01	0.03	0.03	-0.00	0.02	0.02	-0.00	0.02	0.02
	M-spline	-0.00	0.04	0.04	-0.01	0.04	0.04	-0.00	0.05	0.05
O	Cox ROC	-0.21	0.00	0.21	-0.08	0.03	0.08	-0.06	0.03	0.06
	Cox prob	-0.21	0.00	0.21	-0.07	0.02	0.08	-0.06	0.02	0.06
	PW-const	-0.10	0.02	0.10	-0.06	0.02	0.07	-0.05	0.02	0.06
	Weibull	-0.01	0.04	0.04	-0.00	0.03	0.03	0.00	0.03	0.03
	M-spline	-0.07	0.06	0.09	0.00	0.04	0.04	-0.00	0.06	0.06
P	Cox ROC	-0.04	0.02	0.04	-0.02	0.02	0.03	-0.02	0.02	0.03
	Cox prob	-0.04	0.02	0.04	-0.02	0.02	0.03	-0.02	0.02	0.03
	PW-const	-0.05	0.02	0.05	-0.03	0.02	0.03	-0.02	0.02	0.03
	Weibull	-0.00	0.02	0.02	-0.00	0.02	0.02	0.00	0.02	0.02
	M-spline	0.01	0.03	0.03	-0.00	0.04	0.04	-0.00	0.05	0.05
Q	Cox ROC	-0.07	0.03	0.08	-0.04	0.02	0.05	-0.03	0.02	0.04
	Cox prob	-0.07	0.02	0.08	-0.04	0.02	0.04	-0.03	0.02	0.04
	PW-const	-0.07	0.02	0.07	-0.04	0.02	0.04	-0.03	0.02	0.04
	Weibull	-0.01	0.03	0.03	-0.00	0.02	0.02	0.00	0.02	0.02
	M-spline	-0.01	0.04	0.04	-0.00	0.04	0.04	-0.00	0.05	0.05
R	Cox ROC	-0.21	0.00	0.21	-0.07	0.03	0.08	-0.06	0.03	0.06
	Cox prob	-0.21	0.00	0.21	-0.07	0.02	0.08	-0.06	0.02	0.06
	PW-const	-0.10	0.02	0.10	-0.06	0.02	0.07	-0.05	0.02	0.06
	Weibull	-0.01	0.04	0.04	-0.00	0.02	0.02	-0.00	0.02	0.02
	M-spline	-0.06	0.06	0.09	0.00	0.05	0.05	0.00	0.06	0.06

Abbreviations: $\text{AUC}^{\text{I/D}}(t)$, incident/dynamic AUC at year t; PW-const, piecewise-constant model; *Cox ROC* estimates are obtained via the `risksetAUC` function from the `risksetROC` package; *Cox prob* estimates are based on transition probabilities of Cox model through `mstate` package.

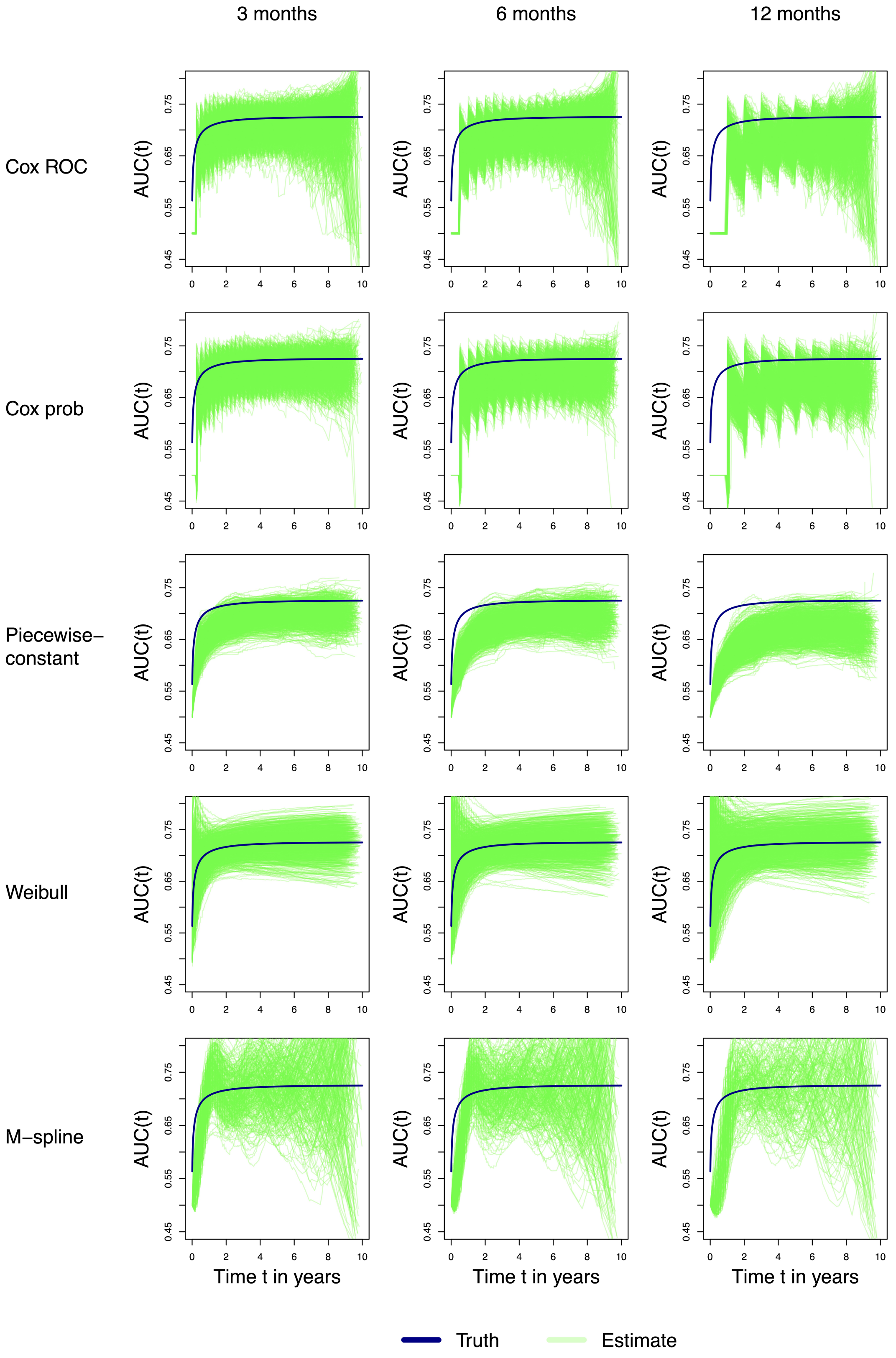

Figure C.7: Estimated time-specific incident/dynamic AUC for scenario M (3 months; left panels), N (6 months; middle panels) and O (12 months; right panels) using different models (Cox, PW-const, Weibull, M-spline). The x-axis represents time t in years; the y-axis represents $\widehat{\mathrm{AUC}}^{\mathrm{I/D}}(t)$. The blue line in each panel represents the true values over time. Estimates for *Cox ROC* (first row) are based on `risksetAUC` function for Cox model. Estimates for *Cox prob* are based on transition probabilities of Cox model via `mstate` package.

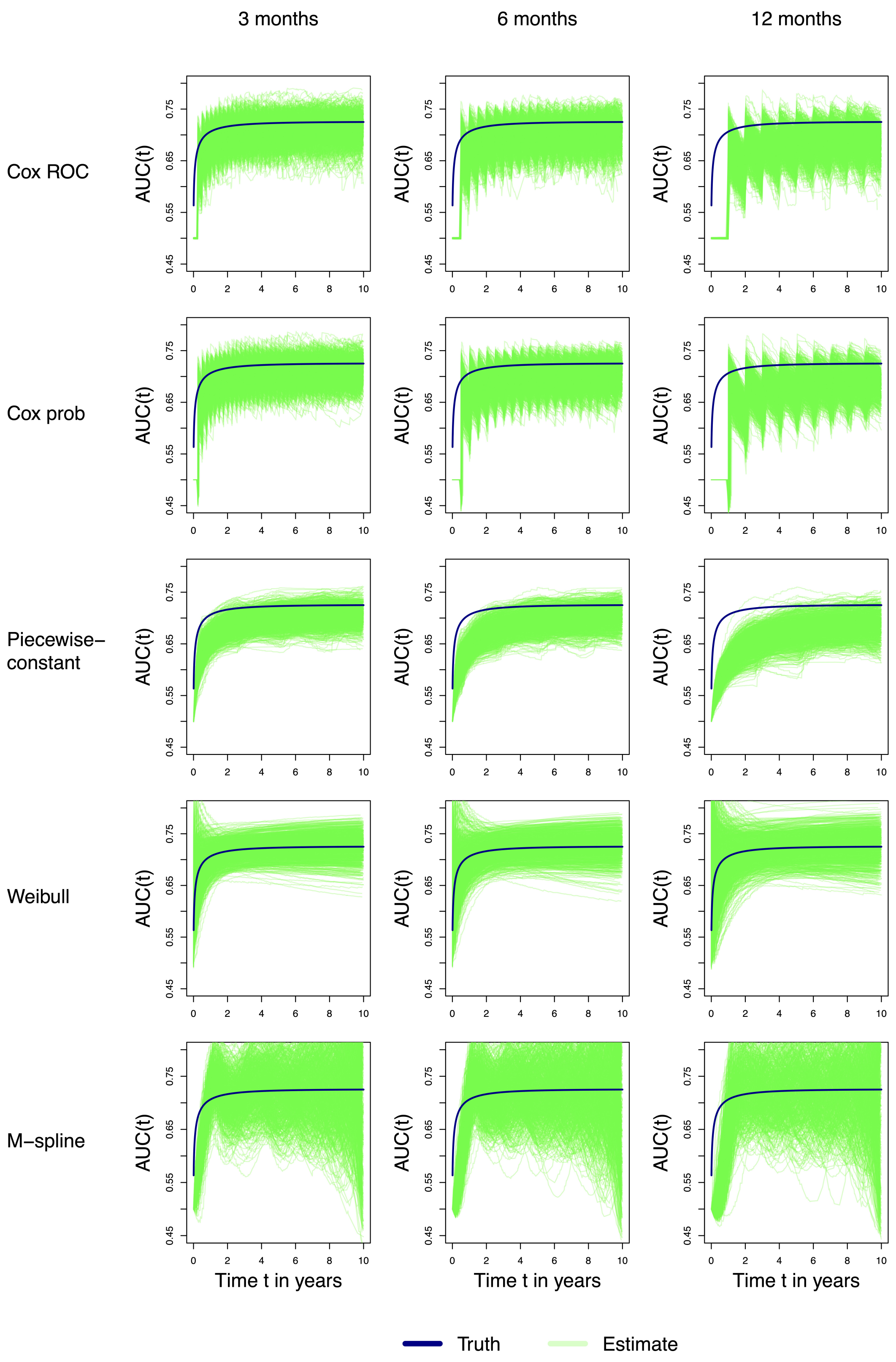

Figure C.8: Estimated time-specific incident/dynamic AUC for scenario G (3 months; left panels), H (6 months; middle panels) and I (12 months; right panels) using different models (Cox, PW-const, Weibull, M-spline). The x-axis represents time t in years; the y-axis represents $\widehat{\text{AUC}}^{\text{I/D}}(t)$. The blue line in each panel represents the true values over time. Estimates for *Cox ROC* (first row) are based on `risksetAUC` function for Cox model. Estimates for *Cox prob* are based on transition probabilities of Cox model via `mstate` package.

Table C.7: Estimated bias, empirical standard error (SE), and root mean square error (RMSE) for time-specific cumulative/dynamic AUC for prediction time 1, 3, 5 years and prediction window of 5 years under scenarios M to R.

		$\text{AUC}^{C/D}(1,6) = 0.59$			$\text{AUC}^{C/D}(3,7) = 0.62$			$\text{AUC}^{C/D}(5,8) = 0.64$		
Scenario	Model	Bias	SE	RMSE	Bias	SE	RMSE	Bias	SE	RMSE
M	Cox prob	-0.00	0.02	0.02	-0.00	0.02	0.02	0.00	0.02	0.02
	PW-const	-0.02	0.01	0.02	-0.01	0.01	0.02	-0.01	0.02	0.02
	Weibull	-0.00	0.01	0.01	-0.00	0.01	0.01	-0.00	0.01	0.01
	M-spline	0.01	0.02	0.02	0.00	0.02	0.02	-0.00	0.03	0.03
N	Cox prob	0.00	0.02	0.02	-0.00	0.02	0.02	0.00	0.02	0.02
	PW-const	-0.04	0.01	0.04	-0.02	0.01	0.03	-0.02	0.02	0.03
	Weibull	-0.01	0.01	0.01	-0.00	0.01	0.01	-0.00	0.01	0.01
	M-spline	0.00	0.02	0.02	-0.00	0.02	0.02	-0.01	0.03	0.03
O	Cox prob	0.00	0.02	0.02	-0.00	0.02	0.02	0.00	0.02	0.02
	PW-const	-0.05	0.01	0.05	-0.04	0.01	0.04	-0.03	0.02	0.04
	Weibull	-0.01	0.01	0.01	-0.00	0.01	0.01	-0.00	0.01	0.01
	M-spline	-0.00	0.02	0.02	-0.00	0.02	0.02	-0.01	0.03	0.03
P	Cox prob	-0.00	0.01	0.01	0.00	0.02	0.02	-0.00	0.02	0.02
	PW-const	-0.02	0.01	0.02	-0.01	0.01	0.02	-0.01	0.02	0.02
	Weibull	-0.00	0.01	0.01	-0.00	0.01	0.01	-0.00	0.01	0.01
	M-spline	0.00	0.01	0.01	0.00	0.02	0.02	-0.00	0.02	0.02
Q	Cox prob	0.00	0.02	0.02	-0.00	0.02	0.02	-0.00	0.02	0.02
	PW-const	-0.04	0.01	0.04	-0.03	0.01	0.03	-0.02	0.02	0.03
	Weibull	-0.00	0.01	0.01	-0.00	0.01	0.01	-0.00	0.01	0.01
	M-spline	0.00	0.02	0.02	-0.00	0.02	0.02	-0.00	0.02	0.02
R	Cox prob	0.00	0.02	0.02	-0.00	0.02	0.02	-0.00	0.02	0.02
	PW-const	-0.05	0.01	0.05	-0.04	0.01	0.04	-0.03	0.02	0.04
	Weibull	-0.01	0.01	0.01	-0.00	0.01	0.01	-0.00	0.01	0.01
	M-spline	-0.00	0.02	0.02	-0.00	0.02	0.02	0.00	0.03	0.03

Abbreviations: $\text{AUC}^{C/D}(t, t+5)$, cumulative/dynamic AUC for prediction time t in years and prediction window of 5 years

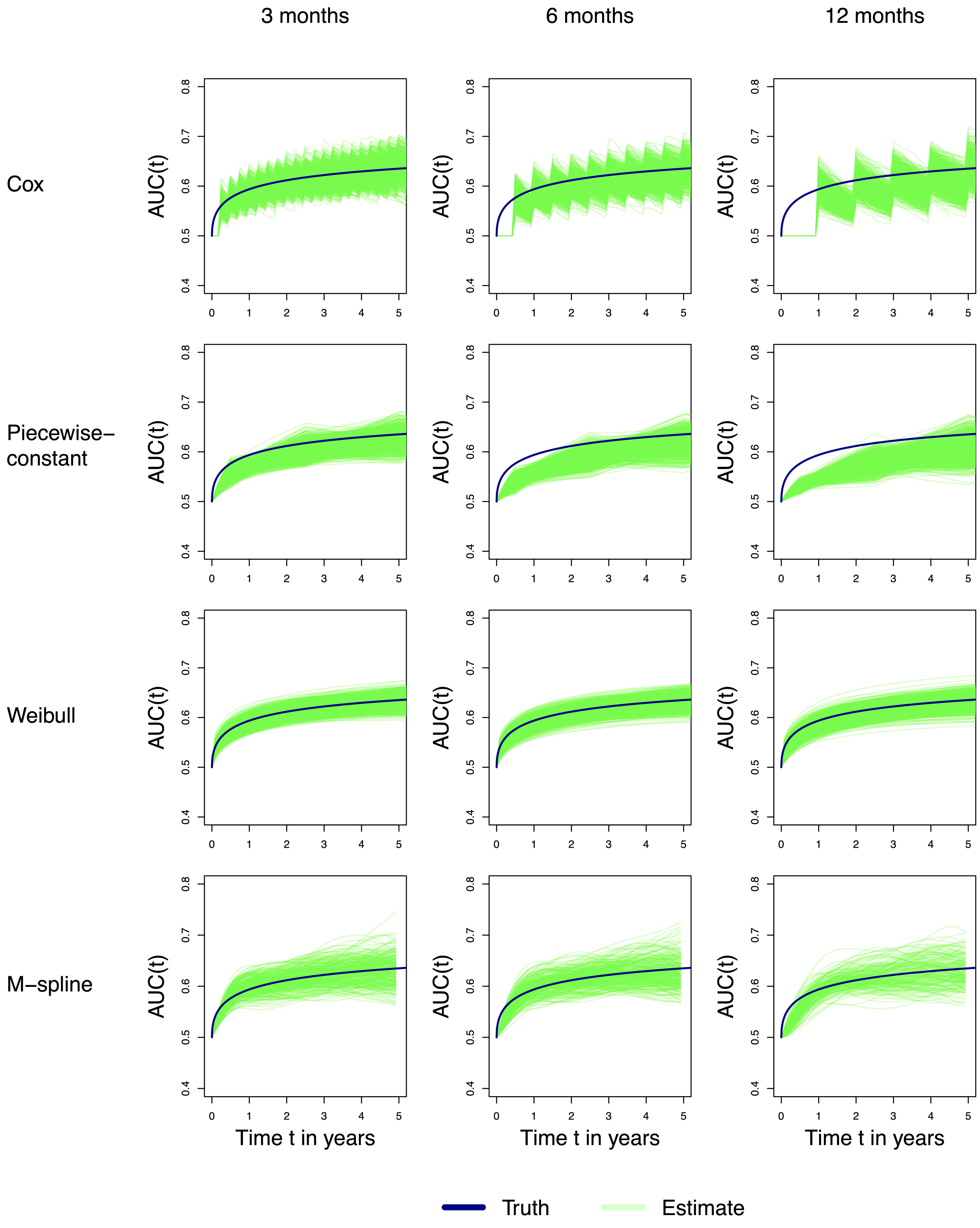

Figure C.9: Estimated time-specific cumulative/dynamic AUC for scenario M (3 months; left panels), N (6 months; middle panels) and O (12 months; right panels) using different models (Cox, PW-const, Weibull, M-spline). The x-axis represents the prediction time t in years. The prediction window is set to 5 years, so the y-axis represents $\widehat{\text{AUC}}^{\text{C/D}}(t, t+5)$. The blue line in each panel represents the true values over time.

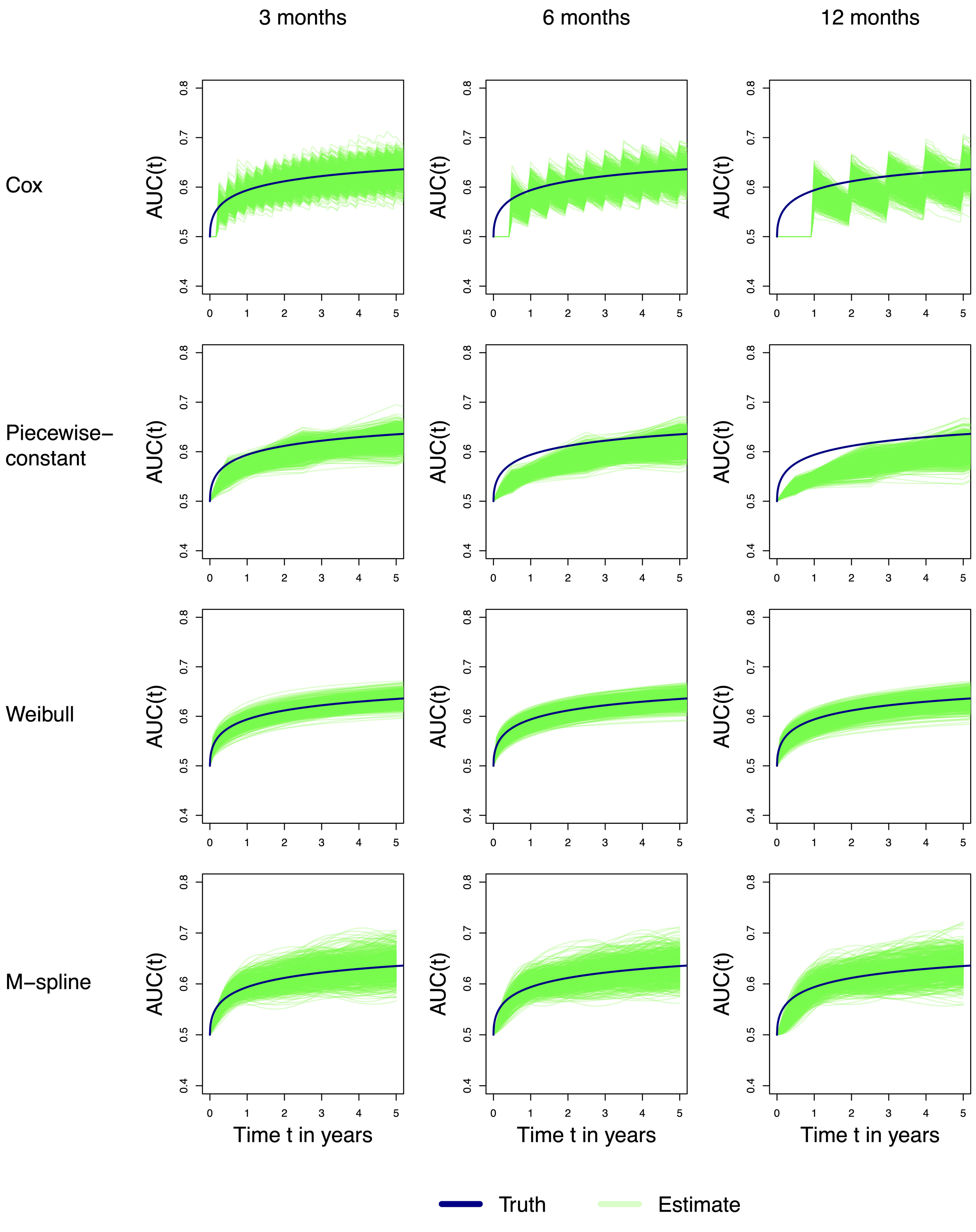

Figure C.10: Estimated time-specific cumulative/dynamic AUC for scenario P (3 months; left panels), Q (6 months; middle panels) and R (12 months; right panels) using different models (Cox, PW-const, Weibull, M-spline). The x-axis represents the prediction time t in years. The prediction window is set to 5 years, so the y-axis represents $\widehat{\mathrm{AUC}}^{\mathrm{C/D}}(t, t+5)$. The blue line in each panel represents the true values over time.
